# Editorial: Children's drawings: evidence-based research and practice

**DOI:** 10.3389/fpsyg.2023.1250556

**Published:** 2023-07-18

**Authors:** Matteo Angelo Fabris, Christiane Lange-Küttner, Monica Shiakou, Claudio Longobardi

**Affiliations:** ^1^Department of Psychology, University of Turin, Turin, Italy; ^2^Leibniz Institute for Educational Trajectories, Bamberg, Germany; ^3^Department of Social and Behavioural Sciences, European University Cyprus, Nicosia, Cyprus

**Keywords:** children's drawing, student-teacher relationship, psychological wellbeing, emotional and social development, pictorial space

Drawing is one of the few surviving forms of human expression from prehistoric times (Lange-Küttner, 2020). It is also considered an enjoyable, playful, and entertaining activity for children and represents an important means of non-verbal communication for them (Longobardi et al., 2022). For this reason, children's drawings have attracted the interest of professionals working with children. The developmental psychologists Piaget and Inhelder (1956) used the technical details of drawings to decipher children's space concepts, while Goodenough (1926) counted details and found them to correlate with IQ. Psychoanalysts have tried to interpret children's psychological dynamics through the graphic characteristics and symbolic contents of their drawings (Kramer, 1979). In general, it is believed that drawing can be a window into the child's inner world, allowing to capture feelings, representations, and perceptions related to a specific topic of investigation (Bozzato et al., 2021; Kallitsoglou et al., 2022). Therefore, it is considered that through drawing, the child does not represent a realistic copy of the external world but rather what they feel, think, and know about the surrounding reality (Quaglia et al., 2015).

Although interest in children's drawings has existed since the 19^th^ century, research on the development of drawing and its potential is more recent. There is a need to increase scientific inquiry into the potential applications of children's drawings in prevention, assessment, and treatment interventions in various developmental contexts. Although research has moved in this direction, more work is still needed, particularly to overcome the various methodological limitations that characterize the current literature.

This Research Topic collected 17 publications from different cultural contexts, 14 of which are research articles. Each of these publications has the merit of increasing our knowledge of the potential and reliability of analyzing children's drawings, both as a research tool and for assessing children's psychological adjustment.

## Drawing as a tool for assessing a child's knowledge, attitudes, and beliefs

Two studies address the use of drawings for assessing children's attitudes and knowledge about Research Topics. The study by Brechet et al. sought to examine the development of children's knowledge of the brain and its functioning. Data from a sample of more than 250 French elementary school students showed that their drawings tended to reflect their current knowledge of how the 'black box' of the brain works. Graphical indicators of knowledge about brain functioning became more frequent with age. These data are important because they underscore the usefulness of drawings in assessing children's metacognitive knowledge and remind us of the importance of neuroeducation in elementary school. Profice et al. used the design to examine the effectiveness of an environmental education program (BioBrasil) implemented with adolescents to develop their knowledge and attitudes about nature. The authors found that direct contact with nature favors consolidation of knowledge and greater closeness to the natural environment visited. They also seem to suggest that drawing is a valuable tool for understanding adolescents' attitudes toward nature.

## Drawing as a tool to investigate the child's representations

Some research has used drawing to examine children's representations of social phenomena, activities, and the physical contexts in which they are embedded, primarily the school environment. Marengo et al. conducted an interesting mixed-methods study using interviews and drawings to analyze descriptions of bullying among more than 600 elementary school children. Interestingly, the experience of being involved in bullying, whether as a victim, bully, or as a bully and victim simultaneously, was reflected in the children's drawings when they depicted the concept of bullying. In addition, the authors pointed out that interviews provide a more comprehensive and general insight into children's portrayal of bullying, while the drawings illustrate children's personal experiences of bullying.

In another Italian study, Berti and Cigala proposed a new instrument to assess children's representation of the preschool environme nt: DRAW.IN.G. (DRAWing and Interview Grid). The instrument consists of five main dimensions of children's representations of the educational environment—physical, behavioral, relational, emotional, and motivational—in 18 macro-categories and 90 categories that make up the scoring grid. This study involved 262 Italian pre-school children who were asked to draw their favorite place at school. Although validation studies indicate the potential of this method, some critical aspects have emerged that the authors urge us to consider.

In Germany, Rott et al. proposed the development of the Draw a Mathematics Classroom test in order to assess elementary students' representations of their mathematics lessons in classrooms. The authors focused on developing and validating coding of the data with low-inference categories. The results confirm the reliability and validity of the methodological approach. The students' drawings suggest that almost half of the participating students perceived their lessons to be teacher centered.

Moreover, Hatisaru, who is interested in the graphic representation of mathematics, proposed a new framework, the legitimacy code theory (LCT), to critically analyze drawing-based research in mathematics education. The author conducted two studies in Ankara, Turkey, involving primary and middle school students. Overall, both studies emphasized the students' perceptions of mathematical content, discipline-related issues, and attitudes toward mathematics and mathematicians. The application of the LCT provides a framework for analyzing and understanding the knowledge produced through drawing-based research in mathematics education.

## Drawing as a tool for investigating children's emotional experience and psychological and relational wellbeing

One of the major uses of drawing is as a tool for assessing the psychological and relational wellbeing of children and adolescents. In clinical and legal settings, the Family Drawing test is among the most widely used projective tests for assessing the quality of a child's family relationships with a scoring system for assessing attachment (Kallitsoglou et al., 2022). In their opinion paper, Pace et al. discussed the strengths and weaknesses of the Family Drawing test with such an attachment-based coding system. The authors discussed salient aspects such as the test's psychometric validity and its use in different cultural contexts while also offering important insights for future research.

Di Norcia et al. used the Pictorial Assessment of Interpersonal Relationships (PAIR) to investigate the quality of the teacher–pupil relationship and school adjustment in primary school students. The authors asked children to draw two situations in which they were involved with a teacher: one situation characterized by distress and the other by wellbeing. Amongst the many results, the authors identified that the authority of the teacher, of which the pictorial valorization is an index, is internalized even by the youngest children and does not vary in a stressful situation vs. a wellbeing situation.

Also addressing the school context is the Italian contribution of La Grutta et al., who recruited some 1,700 primary and secondary school children. The authors used the “Drawn Stories Technique” and the “Classroom Drawing” to assess children's emotional state within the class group and their scholastic integration in an educational context. The authors found some significant age and gender differences. Furthermore, they recommended the use of the drawing technique to facilitate dialogue with children, modulate didactical materials, and detect and prevent some problems in group class functioning.

Two Italian studies focused on analyzing the emotional experiences of children and adolescents during the COVID-19 pandemic through drawing. Cornaggia et al. surveyed a small sample of 18 elementary school children and asked them to draw three moments: “Before” the pandemic and “During” and “After” the lockdown. According to the authors, it appeared that the children felt sufficiently capable of coping with the situation, as evidenced by the fact that they included themselves in the drawings and indicated many details of their houses in the “During” drawings. However, a sense of loneliness and lack of friends also emerged from the representations, as evidenced by the fact that the children depicted significantly more friends in the drawings that concerned the future.

In the second study, Capurso et al. recruited 900 children and preadolescents (aged 7–13) who were asked to draw a moment in their lives during lockdown. The authors reported a detailed qualitative and quantitative analysis that yielded interesting data. According to the authors, children coped with the lockdown through play, screen use, and technology use. However, the high incidence of lack of self-expression found among preschoolers may indicate how enforced solitude and lack of direct physical contact with others affected their self-perception.

Considering the impact that adverse developmental experiences can have on the psychological development of individuals, Ballús et al. sought to investigate whether graphic emotional indicators were expressed in drawings of the projective Draw-a-Person test made by children in dangerous or neglectful situations. The results of this Spanish study, conducted on children and pre-adolescents, show a high frequency of graphic indicators that are often associated in the literature with experiences of abuse and maltreatment. According to the authors, this is an important finding because it would support the usefulness of drawing human figures in identifying children at risk of victimization.

## Drawing as a learning strategy

In Estonia, Tolsberg et al. have revealed some evidence of the effectiveness of the “Learning with Understanding” program that instructs teachers to help children to develop metacognitive awareness of learning strategies, including drawing, in order to better process study material. Drawing is therefore considered a constructive learning strategy. The authors suggested the importance of the use of schematic drawings in learning processes and provided important evidence on the usefulness of drawings in training programs for teachers.

## The role of possible cultural influences

One of the long-standing questions related to research in the field of children's drawings is the possible influence of the cultural environment. The work by Restoy et al., which attempts to analyze possible cultural influences on the self-portraits of children and adolescents, is very interesting in particular. For the study, 958 self-portraits of children aged 2 to 15 years from 35 different countries on five continents were used. The authors found the existence of cultural variations in the self-portrait patterns. In addition, they found how age and physical vs. sociocultural context may influence self-portrait drawing. In particular, they found an influence of the physical and socio-cultural contexts through the level of urbanization and the degree of individualism of the countries, which affected the complexity, content, and representation of human figures in the observed drawings.

## Children's drawings: a look at developmental processes and research methodology

Research on children's drawings, however, is not limited to the extent to which drawing may be an expression of a child's mental state, perceptions, or world knowledge. More research is needed on the developmental processes of drawing as a process, i.e., the cognitive factors underlying the capacity for visually realistic representation. Lange-Küttner and Vinueza Chavez have made an interesting empirical contribution in this direction to the Research Topic. Through an innovative experimental design, using real spatial models and recording the drawings online, the authors aimed to test whether a negative space drawing technique could help children to draw in perspective. In a sample of five age groups from 5 up to 12 years plus adults, the negative space technique was understood and used only from the age of 9. This work makes a valuable contribution to the long-standing debate in developmental psychology about intellectual and visual realism in children's drawings and to the object- and space-based distinction of attention in cognitive psychology.

From a methodological perspective, the contribution of Jensen et al. shows the importance of using new technologies in the analysis of drawings. They scanned children's drawings and fed them into a machine learning algorithm that would classify selected drawing features into classes. To compare, human evaluations were collected. The authors pointed out that machine and human metrics capture different aspects of the structure of drawings and are both independently useful for evaluating and predicting participants' drawing characteristics.

In addition, Beltzung et al. presented a detailed literature review of deep learning applied to the study of drawings and provided a list of drawing datasets relevant to deep learning approaches. The authors aimed to offer an overview of how deep learning has been and can be used to improve our understanding of drawing behavior. The authors pointed out that both traditional and comparative cognitive methods used in psychology to analyze drawings rely on the subjective interpretation of the experimenter, which can limit the reproducibility of the results. According to the authors, deep learning could contribute to solving this problem.

To summarize, the research and theoretical contributions assembled in this Research Topic demonstrate the potential of children's drawings as a research and assessment tool for children and adolescents. However, much research is still needed to understand the cognitive and neurobiological factors underlying the development of child drawing and to explore its applicability in a variety of assessment and research contexts in the field of developmental psychology. Future research may increasingly face the development of new artificial intelligence (AI) technologies to develop new analytical tools.

Moreover, there is still a need to overcome and clarify the limitations that currently impact empirical research on children's drawings. These include the psychometric properties of instruments used for the quantitative analysis of drawings, the role of the developmental level of graphic ability, the influence of cultural variables, and the generalizability of results. It is our hope that this Research Topic will encourage researchers to improve the quality of research in this area and further investigate the development of drawing and its potential in assessment, prevention, and treatment interventions, as well as an investigative tool in empirical and experimental research.

## Author contributions

All authors listed have made a substantial, direct, and intellectual contribution to the work and approved it for publication.
